# HaploJuice : accurate haplotype assembly from a pool of sequences with known relative concentrations

**DOI:** 10.1186/s12859-018-2424-7

**Published:** 2018-10-22

**Authors:** Thomas K. F. Wong, Louis Ranjard, Yu Lin, Allen G. Rodrigo

**Affiliations:** 1 The Research School of Biology, The Australian National University, Acton ACT, 2601 Australia; 2 College of Engineering and Computer Science, The Australian National University, Acton ACT, 2601 Australia

**Keywords:** Pooling strategy, Haplotype reconstruction, Barcode

## Abstract

**Background:**

Pooling techniques, where multiple sub-samples are mixed in a single sample, are widely used to take full advantage of high-throughput DNA sequencing. Recently, Ranjard et al. (PLoS ONE 13:0195090, 2018) proposed a pooling strategy without the use of barcodes. Three sub-samples were mixed in different known proportions (i.e. 62.5%, 25% and 12.5%), and a method was developed to use these proportions to reconstruct the three haplotypes effectively.

**Results:**

HaploJuice provides an alternative haplotype reconstruction algorithm for Ranjard et al.’s pooling strategy. HaploJuice significantly increases the accuracy by first identifying the empirical proportions of the three mixed sub-samples and then assembling the haplotypes using a dynamic programming approach. HaploJuice was evaluated against five different assembly algorithms, Hmmfreq (Ranjard et al., PLoS ONE 13:0195090, 2018), ShoRAH (Zagordi et al., BMC Bioinformatics 12:119, 2011), SAVAGE (Baaijens et al., Genome Res 27:835-848, 2017), PredictHaplo (Prabhakaran et al., IEEE/ACM Trans Comput Biol Bioinform 11:182-91, 2014) and QuRe (Prosperi and Salemi, Bioinformatics 28:132-3, 2012). Using simulated and real data sets, HaploJuice reconstructed the true sequences with the highest coverage and the lowest error rate.

**Conclusion:**

HaploJuice provides high accuracy in haplotype reconstruction, making Ranjard et al.’s pooling strategy more efficient, feasible, and applicable, with the benefit of reducing the sequencing cost.

## Background

With the rapid advancement of next-generation sequencing technologies, it is possible to obtain several gigabases of sequences in a single day. Given the huge volume of throughput, it is often cost-effective to mix multiple sub-samples in a single sample for sequencing, a process called pooling. Several approaches have been developed to demultiplex the sequencing reads from the mixture, i.e. assign reads to their respective sub-samples. For example, a short unique identifiable sequence tag (i.e. barcode) is often appended to each DNA molecule of the same sub-sample before pooling and sequencing. Barcodes allow the reads to be separated into different groups according to their unique barcode sequences [1]. Each group is expected to originate from the same individual as with unpooled samples. Individual haplotypes can then be reconstructed by either by de novo assembly or computing the consensus sequence after aligning reads against one or more reference sequences. This approach cannot be applied to a mixture of reads without barcodes because the reads cannot be demultiplexed.

Nonetheless, in some instances, it may be useful to recover the constituent haplotype sequences from a mixture of haplotypes without using barcodes because the cost of the library preparation increases linearly with the number of required barcodes. Therefore, if it is possible to efficiently reconstruct haplotypes from mixtures of samples without using barcodes, this may reduce sequencing costs significantly.

Several methods have been designed to reconstruct the haplotypes from a mixture of reads without barcodes. The simplest of these approaches, developed by [2], aligns a mixture of reads against several reference sequences, allowing them to separate the reads to the different references. However, their method is only applicable for samples which are phylogenetically distant enough, e.g., for different species.

More sophisticated methods have also been developed to recover the constituent sequences from mixtures, when these sequences are genetically quite similar, e.g., haplotypes within populations or species. ShoRAH [3] implements local-window clustering to recover the constituent haplotypes in a mixture. SAVAGE [4] uses an overlap graph and clique enumeration to reconstruct multiple haplotypes. PredictHaplo [5] uses Dirichlet prior mixture model, starts local reconstruction at the region of maximum coverage and progressively increases the region size until it covers the entire length of haplotypes. QuRe [6] uses sliding windows and reconstructs the haplotypes based on multinomial distribution matching heuristic algorithm [7]. However, ShoRAH, SAVAGE, PredictHaplo and QuRe assume that both the number and the proportion of the constituent haplotypes in the mixture are unknown and do not make use of these information in their algorithms.

Recently, Ranjard, et al. [8] proposed another pooling strategy without barcodes that can be applied for individuals of the same species. Their strategy consists of pooling in a single sample, individually amplified sequences in different known proportions. The proportions of these ‘sub-samples’ induce different expected frequencies of the variants in the mixture, and hence, different expected sequencing read coverages. These frequencies, in turn, allow the sub-sampled sequences to be reconstructed accurately. Ranjard et al. applied their method to mitochondrial sequences from three kangaroo sub-samples (each sub-sample consisting of an amplified fragment from a single kangaroo) mixed in proportions 62.5%, 25%, and 12.5%, and showed that the three haplotypes could be assembled effectively, thus reducing the cost of sequencing significantly. Hmmfreq [8], which was developed by Ranjard et al. to reconstruct the haplotypes under this scenario, is based on a Dirichlet-multinomial model [9] and a Hidden Markov Model (HMM).

In this paper, we focus on the pooling strategy [8] proposed by Ranjard et al. but our method, however, does not assume any prior knowledge on the sample proportions; only the number of sub-samples in the mixture is known a priori. We compute the sub-sample proportions directly from the mixture of reads using a maximum likelihood method. Based on the estimated sample proportions, we use a multinomial model and dynamic programming to reconstruct the multiple haplotypes simultaneously.

HaploJuice, which is an extension of Hmmfreq [8], considers all possible combinations for assigning local sub-sequences to haplotypes, and selects the combination with the highest overall likelihood. We evaluate HaploJuice against five different assembly algorithms, Hmmfreq [8], ShoRAH [3], SAVAGE [4], PredictHaplo [5] and QuRe [6], using simulated and real data sets in which three sequences are mixed in known frequencies. Based on our results, HaploJuice reconstructs sequences with the highest coverage of the true sequences and has the lowest error rate.

## Results

HaploJuice first identifies the underlying sub-sample proportions from a mixture of reads and, second, reconstructs the haplotypes using these estimated proportions. As with Hmmfreq it requires an alignment of short-read sequences against a reference sequence. In our analysis, all reads are aligned to the reference sequence using Bowtie 2 [10].

Simulated datasets were used to evaluate our methods. Four hundred data sets were simulated and each data set was a mixture of three sub-samples. The three sub-samples were mixed under various proportions: 5:4:1, 5:3:2, 6:3:1, and 7:2:1 (100 data sets each). 150-long pair-ended reads with total coverage 1500x were simulated by ART [11] with the default Illumina error model from three 10k-long haplotypes, which were generated by INDELible [12] using JC [13] model from a 3-tipped tree with 0.05 root-to-tip distance randomly created by Evolver [14] from PAML [15] package.

After using Bowtie 2 [10] to align the reads against the root sequence (also reported from INDELible [12]), we ran HaploJuice to estimate the sub-sample proportions in the mixture. As shown in Table 1, on average, the estimated sub-sample proportions were the same as the actual proportions with standard deviation 0.001. The method of estimation on the sub-sample proportions is, therefore, found to be effective on these simulated data sets.

**Table 1:** The results of estimation on the sample proportions by HaploJuice

Case	Actual sample proportion			Estimated sample proportion		
	*f*_1_	*f*_2_	*f*_3_	(Average ± Standard deviation)		
1	0.5	0.4	0.1	0.50 ± 0.001	0.40 ± 0.001	0.10 ± 0.001
2	0.5	0.3	0.2	0.50 ± 0.001	0.30 ± 0.001	0.20 ± 0.001
3	0.6	0.3	0.1	0.60 ± 0.001	0.30 ± 0.001	0.10 ± 0.001
4	0.7	0.2	0.1	0.70 ± 0.001	0.20 ± 0.001	0.10 ± 0.001

One hundred data sets were simulated for each case

HaploJuice was then used to reconstruct the haplotype sequences for each data set based on the estimated sample proportions. HaploJuice was compared to five different assembly algorithms, including Hmmfreq [8], ShoRAH [3], SAVAGE [4], PredictHaplo [5] and QuRe [6]. Note that SAVAGE, PredictHaplo and QuRe do not have prior assumptions on the number of haplotypes, whereas HaploJuice and Hmmfreq do. MetaQUAST [16] was then used with default parameters to evaluate the contigs, which were resulted by all the software, against the true sequences. By default, MetaQUAST discards all the contigs with length smaller than 500. Table 2 shows the summary of the performance of different methods on the simulated data sets. On average, HaploJuice reconstructed contigs over 99.7% haplotype coverage, which was the highest among all the methods. When checking the error rates (i.e. the percentage of bases in the contig sequences having mutations or indels when compared against with the real haplotypes), HaploJuice was less than 0.005% on average. It was the lowest among the software which reconstructed contigs over 90% haplotype coverage. In conclusion, HaploJuice is shown effective from the simulated data sets.

**Table 2:** Comparison of performance of different methods on reconstruction of three haplotypes for simulated data sets

a. Proportion of three samples: 0.5, 0.4, 0.1 (total length of three haplotypes: 30k)					
Software	# contigs	Longest	N50	Haplotypes	Error rate *%*
	≥ 500 bp	contig		coverage *%*	
HaploJuice	**3.0 ± 0.0**	**9975 ± 6.8**	**9971 ± 6.5**	**99.7 ± 0.0**	**0.001 ± 0.004**
hmmfreq[8]	**3.0 ± 0.0**	9855 ± 6.8	9850 ± 6.3	98.5 ± 0.0	0.276 ± 0.254
shoRAH[3]	30.8 ± 11.7	9819 ± 124.8	9799 ± 116.7	97.5 ± 3.5	0.646 ± 0.492
SAVAGE[4]	9.8 ± 3.5	9972 ± 11.8	305 ± 300.3	51.3 ± 7.1	0.001 ± 0.004
PredictHaplo[5]	2.0 ± 0.2	9991 ± 4.2	9984 ± 5.6	67.7 ± 5.7	0.102 ± 0.034
QuRe[6]	3.7 ± 1.9	6993 ± 1306.3	7374 ± 686.5	43.8 ± 13.5	0.331 ± 0.318
b. Proportion of three samples: 0.5, 0.3, 0.2 (total length of three haplotypes: 30k)					
Software	# contigs	Longest	N50	Haplotypes	Error rate *%*
	≥ 500bp	contig		coverage *%*	
HaploJuice	**3.0 ± 0.0**	**9975 ± 6.3**	**9971 ± 7.8**	**99.7 ± 0.0**	**0.000 ± 0.001**
hmmfreq[8]	**3.0 ± 0.0**	9854 ± 5.8	9850 ± 7.6	98.5 ± 0.0	0.089 ± 0.104
shoRAH[3]	27.9 ± 6.6	9814 ± 118.3	9789 ± 113.9	97.1 ± 4.7	0.591 ± 0.358
SAVAGE[4]	11.4 ± 3.4	9983 ± 8.2	436 ± 281.8	54.7 ± 7.1	0.001 ± 0.005
PredictHaplo[5]	2.0 ± 0.2	9991 ± 3.7	9984 ± 5.8	68.0 ± 6.6	0.087 ± 0.040
QuRe[6]	4.2 ± 2.2	7348 ± 820.8	7436 ± 776.9	44.9 ± 15.9	0.761 ± 0.851
c. Proportion of three samples: 0.6, 0.3, 0.1 (total length of three haplotypes: 30k)					
Software	# contigs	Longest	N50	Haplotypes	Error rate *%*
	≥ 500bp	contig		coverage *%*	
HaploJuice	**3.0 ± 0.0**	**9975 ± 7.3**	**9970 ± 7.7**	**99.7 ± 0.0**	**0.000 ± 0.000**
hmmfreq[8]	**3.0 ± 0.0**	9854 ± 5.6	9849 ± 6.2	98.5 ± 0.0	0.210 ± 0.214
shoRAH[3]	25.2 ± 5.9	9837 ± 115.0	9808 ± 113.3	97.4 ± 4.8	0.749 ± 0.516
SAVAGE[4]	11.2 ± 3.0	9971 ± 20.9	419 ± 260.5	53.9 ± 6.3	0.001 ± 0.006
PredictHaplo[5]	2.0 ± 0.0	9991 ± 3.5	9984 ± 4.7	66.7 ± 0.0	0.089 ± 0.025
QuRe[6]	3.9 ± 1.9	7074 ± 1284.4	7300 ± 716.6	39.1 ± 14.5	0.492 ± 0.597
d. Proportion of three samples: 0.7, 0.2, 0.1 (total length of three haplotypes: 30k)					
Software	# contigs	Longest	N50	Haplotypes	Error rate *%*
	≥ 500bp	contig		coverage *%*	
HaploJuice	**3.0 ± 0.0**	**9976 ± 6.1**	**9971 ± 6.3**	**99.7 ± 0.0**	**0.005 ± 0.048**
hmmfreq[8]	**3.0 ± 0.0**	9855 ± 6.2	9850 ± 6.7	98.5 ± 0.0	0.240 ± 0.220
shoRAH[3]	20.2 ± 4.7	9835 ± 115.0	9812 ± 106.4	93.8 ± 11.2	0.912 ± 0.630
SAVAGE[4]	15.2 ± 3.0	9974 ± 10.6	708 ± 161.7	65.1 ± 7.0	0.001 ± 0.005
PredictHaplo[5]	2.0 ± 0.0	9991 ± 3.8	9984 ± 4.7	66.7 ± 0.0	0.088 ± 0.021
QuRe[6]	3.6 ± 1.8	6787 ± 1333.0	7121 ± 809.6	28.4 ± 11.2	0.319 ± 0.535

One hundred data sets were generated for each of the cases with different sets of sample proportions. Format of the data is: average ± standard deviation. The best value for each column is **highlighted** among the software outputting the contigs over 90% haplotype coverage

Apart from the simulated data sets, mixtures of reads from three kangaroo sub-samples [8] were also used to evaluate the performance of the methods. These reads [8] were obtained by short read sequencing of three mitochondrial amplicons on an Illumina platform. The sub-samples were mixed in the proportions: 0.625, 0.25, and 0.125 during the library preparation, and the total coverage of reads is 1600x. There is a total of 30 data sets; 10 data sets for each amplicon (three amplicons in total).

All the reads were aligned against the corresponding amplicon regions on the reference mitochondrial sequence [17] (Genbank accession number NC_027424) by Bowtie 2 [10]. The alignment file is the input of HaploJuice and the estimated sub-sample proportions are listed in Table 3. Although the sub-samples were intentionally mixed in the proportions 0.625, 0.25 and 0.125, variations on the estimated proportions were noticed. For example, for the data sets of amplicon 3, the estimated proportions were 0.646, 0.251, and 0.103 on average. The variation between the estimated proportions and the expected proportions was 6.2% on average, ranging from 0.3% to 17.9%. This revealed the fact that the actual sub-sample proportions in the mixture may be differ from expectation, when the sub-samples are mixed manually during the library preparation.

**Table 3:** Estimated frequencies of three kangaroo sub-samples among the mixture of reads [8] for three amplicons resulted from our method

Amplicon	Target proportions			Average estimated proportions (average variation in %)		
	*f*_1_	*f*_2_	*f*_3_	*f*_1_	*f*_2_	*f*_3_
Amplicon 1	0.625	0.250	0.125	0.656	0.229	0.115
				(4.9%)	(8.3%)	(8.0%)
Amplicon 2	0.625	0.250	0.125	0.640	0.246	0.114
				(2.4%)	(1.6%)	(8.7%)
Amplicon 3	0.625	0.250	0.125	0.646	0.251	0.103
				(3.4%)	(0.3%)	(17.9%)

It revealed the existence of variations on the ratios of the sub-samples when mixing them during the library preparation. Ten data sets were for each amplicon

HaploJuice as well as the other five methods, including Hmmfreq [8], ShoRAH [3], SAVAGE [4], PredictHaplo [5] and QuRe [6], were used to reconstruct the three haplotypes for each amplicon region from the mixture of kangaroo reads. MetaQUAST [16] with default parameters was used to evaluate the resulting contigs against the true haplotypes inferred by deep sequencing [8]. Table 4 shows the summary on the performance of different methods. On average, HaploJuice resulted in contigs with the highest haplotype coverage for all amplicons (97% for amplicon 2 and over 99% for amplicon 1 and 3) among all the methods, and with the lowest (or one of the lowest) error rate among the methods with contigs over 90% haplotype coverage (on average, 0.05% for amplicon 1, 0.02% for amplicon 2, and 0.01% for amplicon 3). Thus, HaploJuice is shown to be effective at recovering the constituent haplotypes from the real data sets, even though the read coverage in the data sets fluctuates considerably along the mitochondrial genome (as shown in [8]).

**Table 4:** Comparison of performance of different methods on reconstruction of three haplotypes for real kangaroo data sets from the mixture of reads [8] for (a) amplicon 1, (b) amplicon 2, and (c) amplicon 3

a. Amplicon 1 (total length of three haplotypes: 13921)					
Software	# contigs	Longest	N50	Haplotypes	Error rate *%*
	≥ 500bp	contig		coverage *%*	
HaploJuice	**3.0 ± 0.0**	**4613 ± 2.1**	**4612 ± 2.0**	**99.4 ± 0.0**	**0.05 ± 0.07**
hmmfreq[8]	**3.0 ± 0.0**	4485 ± 0.6	4484 ± 0.6	96.6 ± 0.0	0.26 ± 0.10
shoRAH[3]	24.0 ± 2.6	4592 ± 7.0	4592 ± 6.0	95.6 ± 10.4	1.05 ± 0.32
SAVAGE[4]	13.2 ± 2.1	903 ± 132.3	482 ± 169.6	47.3 ± 5.2	0.02 ± 0.04
PredictHaplo[5]	1.1 ± 0.3	4630 ± 2.0	462 ± 1461.3	36.5 ± 10.5	0.01 ± 0.01
QuRe[6]	4.0 ± 1.9	4343 ± 9.9	3909 ± 1373.7	74.9 ± 21.8	0.42 ± 0.32
b. Amplicon 2 (total length of three haplotypes: 12694)					
Software	# contigs	Longest	N50	Haplotypes	Error rate *%*
	≥ 500bp	contig		coverage *%*	
HaploJuice	**3.0 ± 0.0**	**4120 ± 1.5**	**4120 ± 1.5**	**97.4 ± 0.0**	**0.02 ± 0.03**
hmmfreq[8]	**3.0 ± 0.0**	3998 ± 4.0	3998 ± 4.0	94.5 ± 0.1	**0.02 ± 0.01**
shoRAH[3]	24.2 ± 5.7	4119 ± 14.5	4118 ± 12.1	90.8 ± 13.5	0.41 ± 0.48
SAVAGE[4]	8.8 ± 3.8	1806 ± 761.5	572 ± 81.7	50.2 ± 4.7	0.00 ± 0.00
PredictHaplo[5]	2.0 ± 0.0	4140 ± 2.6	4136 ± 0.0	65.2 ± 0.0	0.00 ± 0.00
QuRe[6]	2.4 ± 0.7	3746 ± 4.7	3373 ± 1185.0	38.4 ± 14.3	0.22 ± 0.28
c. Amplicon 3 (total length of three haplotypes: 15391)					
Software	# contigs	Longest	N50	Haplotypes	Error rate *%*
	≥ 500bp	contig		coverage *%*	
HaploJuice	**3.0 ± 0.0**	5116 ± 9.1	**5111 ± 7.7**	**99.6 ± 0.1**	**0.01 ± 0.00**
hmmfreq[8]	**3.0 ± 0.0**	5029 ± 3.1	5027 ± 3.6	98.0 ± 0.1	0.23 ± 0.11
shoRAH[3]	27.6 ± 3.0	**5132 ± 7.1**	5111 ± 7.4	96.3 ± 10.5	1.91 ± 0.44
SAVAGE[4]	11.8 ± 2.3	2510 ± 672	550 ± 40.4	55.6 ± 4.3	0.01 ± 0.01
PredictHaplo[5]	1.6 ± 0.5	5170 ± 3.9	3070 ± 2642.4	53.3 ± 17.2	0.14 ± 0.09
QuRe[6]	3.0 ± 1.1	4567 ± 2.1	4106 ± 1442.7	35.6 ± 12.5	0.25 ± 0.28

There are 10 data sets for each amplicon with total coverage of the reads 1600x. For each data set, the sub-samples were mixed in the proportions: 0.125, 0.25, 0.625. The format of data is: average ± standard deviation. The best value for each column is highlighted among the methods with contigs over 90% coverage on three haplotypes

**Fig. 1:**
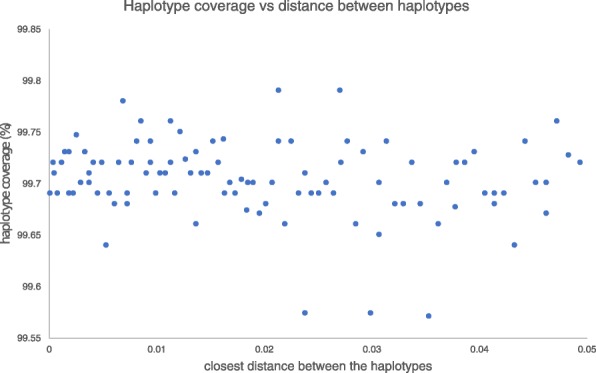
Coverage of HaploJuice contigs as a function of haplotype genetic distances. The figure shows how the performance of HaploJuice varies with different genetic distances between the sub-samples

To understand how the performance of HaploJuice varies with different genetic distances between the sub-samples, another one hundred data sets were simulated. Each data set was a mixture of three sub-samples under the proportions 1:2:5. For each triplet, the root-to-tip genetic distance of the tree was fixed at 0.05, and the genetic distance of the ancestor of the two most closely related sequences was a uniform random variable between 0.001 and 0.05. Similar to the previous simulated data sets, 150-long pair-ended reads with total coverage 1500x were simulated and they were aligned to the root sequence. The haplotype sequences were reconstructed using HaploJuice from the read alignments. Figure 1 shows that the resulting haplotype coverage of the contigs is higher than 99.55% in all data sets, and the resulting error rates of the contigs are less than 0.001% with the exception of in one data set, where the error rate was 0.1% (data not shown). The results indicates that HaploJuice performs consistently with different distances between the haplotypes.

**Fig. 2:**
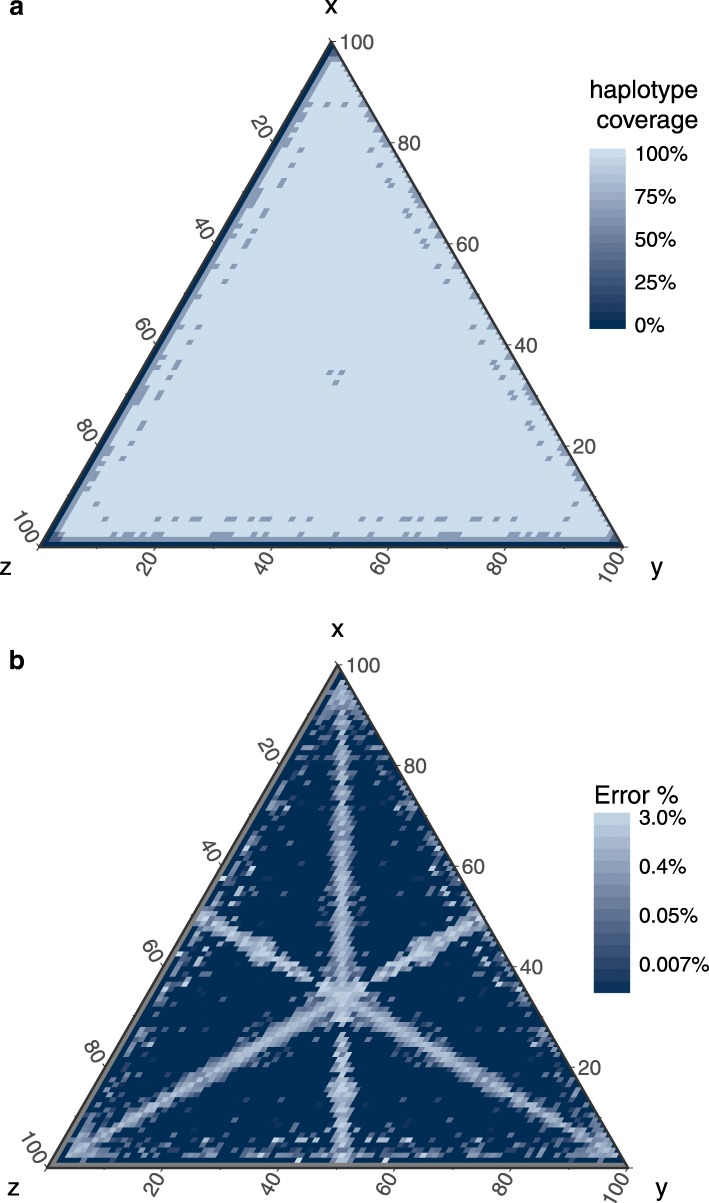
Performance of HaploJuice with different sample frequencies. The figures (**a**) and (**b**) show the haplotype coverages and the error rates of the contigs under different sub-sample proportions, respectively

The performance of HaploJuice was also evaluated under different sub-sample proportions. A total of 833 datasets were simulated to cover all possible unique combinations of three sub-sample proportions with range between 1% and 98%, with a step size of 1%. As before, the 150-long pair-ended reads with total coverage 1500x were simulated and they were aligned to the root sequence. HaploJuice was used to reconstruct the haplotype sequences from the read alignments. Figure 2 shows the performance of HaploJuice with different combinations of sub-sample proportions (i.e. *x*%, *y*%, *z*%). Figure 2a indicates that the haplotype coverage is close to 100%, but decreases when either *x*, *y*, or *z* are too small (i.e. less than 5%). The haplotype coverage also decreases when *x*≈*y*≈*z* (e.g., when sub-sample proportions are 33%, 33%, 34%). Similarly, Fig. 2b shows that the error rates are generally very low, except when two of the sub-sample proportions are close (e.g., *x*≈*y*, *y*≈*z*, *x*≈*z* or *x*≈*y*≈*z*). This result is in line with our expectations, because the algorithm uses proportions to reconstruct haplotypes, and haplotypes having similar proportions will naturally confound the process. From Fig. 2a and b, we found that the haplotype proportions have to be at least 5% different for HaploJuice to perform effectively.

When comparing the running time between different methods on the Kangaroo data sets, HaploJuice was the fastest, averaging 0.14 min for each data set, while other software took from 4 to 139 min. The summary is shown in Table 5.

**Table 5:** The average running time (in min) of different methods to reconstruct haplotypes for each Kangaroo data set

HaploJuice	hmmfreq	ShoRah	SAVAGE	PredictHaplo	QuRe
	[8]	[3]	[4]	[5]	[6]
0.14	13.53	7.81	11.21	4.30	139.93

## Discussion

In order to decrease the cost of sequencing, Ranjard et al. [8] proposed a pooling strategy to mix sub-samples in specific known proportions thus simplifying library preparation by removing the need for barcode sequences. According to their experiments on mitochondrial amplicons from three kangaroo sub-samples mixed in proportions 0.625, 0.25, and 0.125, they found that the three haplotypes could be reconstructed effectively using these known frequencies. However, they found that variation of the ratios of sub-samples when mixing due to stochastic experimental effects can decrease the accuracy of haplotype reconstruction. Our research provides an alternative haplotype reconstruction algorithm for Ranjard et al.’s pooling strategy. We show that estimating the empirical proportions of the mixed sub-samples, prior to the reconstruction the haplotype sequences, significantly increases the accuracy of the approach. As shown from the simulated data sets and the real data sets, our method can, first, accurately identify the underlying sub-sample proportions from a mixture of reads and, second, reconstruct the haplotypes according to these estimated proportions.

The pooling strategy can be applied on a greater number of sequences. Consider a total of *n* sub-samples. A group of three sub-samples of the same species can be mixed in the specific known proportions and applied the same barcode. Thus only $\frac {n}{3}$ barcodes are required and the cost of the library preparation can be greatly reduced. After sequencing, HaploJuice can be used to assemble the reads associated with the same barcode and reconstruct the three haplotypes for each group of the sub-samples. As shown from the simulated data sets and the real data sets, the high accuracy of assembled haplotypes makes the suggested pooling strategy [8] become more realistic, feasible, and applicable.

Our method relies on aligning reads against a reference sequence. The accuracy of the read alignments affects the effectiveness of our method. In our evaluations, we only used alignments reported by Bowtie 2 [10] with mapping quality of at least 20. Whereas we understand that coverage varies along the haplotype, but we assume that ratios of the read coverage for each haplotype at each location follows the same multinomial distribution. If a region on some haplotypes is very different from the reference sequence, reads from this region may not align to the reference, and the induced read coverage for those haplotypes may decrease substantially. The bias in the induced read coverage ratio can cause misleading results, because of its deviation from the common multinomial distribution. Therefore, this method is designed for the pooling strategy applied on the sub-samples that align well with the reference sequence.

HaploJuice assumes that the number of haplotypes is known in advance. There is no equivalent assumption with ShoRAH [3], SAVAGE [4], PredictHaplo [5] and QuRe [6]. Nonetheless, these are the only available software for haplotype reconstruction from a pool of reads originating from a mixture of different sub-samples. We expect that the effectiveness of haplotype reconstruction using these methods are also likely to be improved if the number of haplotypes is known in advance. One reasonable approach to assemble the reads from a sample with unknown number of haplotypes is therefore to develop a statistical method to estimate the number of haplotypes from a mixture of reads, and then reconstruct the haplotypes using our method according to this estimated number of haplotypes.

## Conclusions

HaploJuice is designed for the reconstruction of three pooled haplotypes from a mixture of short sequencing reads obtained under the strategy proposed by Ranjard et al. [8]. As shown from the simulated data sets and the real data sets, HaploJuice provides high accuracy in haplotype reconstruction, thus increasing the estimation efficiency of Ranjard et al.’s pooling strategy.

## Methods

**Fig. 3:**
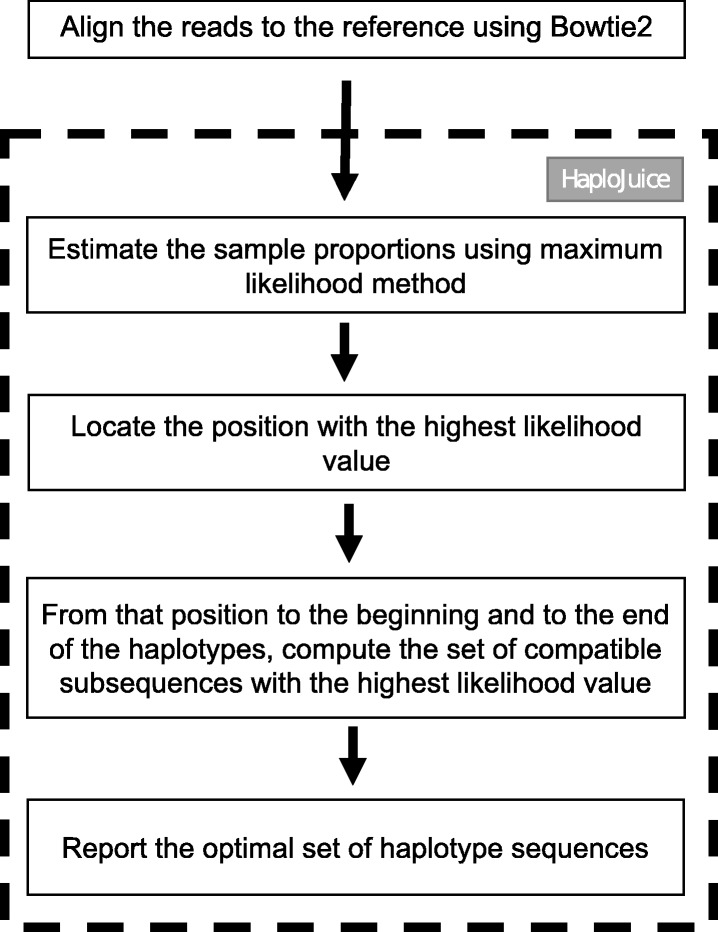
Work flow in HaploJuice. HaploJuice first estimates the sub-sample proportions from a mixture of reads using maximum likelihood method. The algorithm then reconstructs the haplotype sequences using a dynamic programming method

HaploJuice is designed for the pooling strategy [8] proposed by Ranjard et al., assuming the number of sub-samples is known and the sub-samples have different proportions. Figure 3 shows the work flow in HaploJuice. HaploJuice first estimates the sub-sample proportions from a mixture of reads using maximum likelihood method. The algorithm then reconstructs the haplotype sequences using a dynamic programming method. The following subsections describes the details of the algorithm.

### Estimation of sample proportions

HaploJuice requires an alignment of short-read sequences against a reference sequence. All reads are aligned to the reference sequence using Bowtie 2 [10]. Only the reads which are aligned at unique positions on the reference are considered. The alignment of each read has a starting and an ending position on the reference. A sliding window approach is used.

Let *W* be the set of overlapping windows. For each window *w*∈*W*, we collect the reads that are aligned across the whole window. We extract the corresponding sub-sequences according to the window’s bounds, and obtain the set of unique sub-sequences *T*_*w*_={*t*_*w*1_,*t*_*w*2_,...} and the frequencies *G*_*w*_={*g*_*w*1_,*g*_*w*2_,...} where *g*_*wi*_ is the number of reads with subsequence *t*_*wi*_. The sub-sequences inside *T*_*w*_ are sorted in decreasing order of frequencies.

Say *n* sub-samples are pooled with unknown proportions *f*_1_,*f*_2_,...,*f*_*n*_ where *f*_1_>*f*_2_>...>*f*_*n*_. When there is no sequencing error and each sub-sample is from a unique haploid sequence, each sub-sample should produce only one subsequence in *T*_*w*_. In those regions where two or more sub-samples are identical, the sub-sequences originating from these sub-samples will be the same. For each sliding window, the number of possible combinations of *n* samples producing sub-sequences, i.e. the number of possible partitions of a set with *n* different elements (where each element represents a sub-sample, and the elements in the same partition are regarded as the sub-samples producing the same sub-sequences), is the Bell number *B*_*n*_ [18]. Each case will lead to different expected frequencies of the sub-sequences.

However, under real sequencing conditions, the number of sub-sequences in each window may be greater than *n*, because some erroneous sub-sequences are created by sequencing errors. We assume that the frequencies of erroneous sub-sequences are always lower than that of real sub-sequences. For each window, we only consider the top-n most frequent sub-sequences. Table 6 lists the expected frequencies of the sub-sequences for all cases when *n*=3.

**Table 6:** The expected frequencies of top-*n* most frequent sub-sequences for a mixture from 3 samples

Case	Expected frequencies of sub-sequences		
1	*f*_1_	*f*_2_	*f*_3_
2	*f*_1_+*f*_2_	*f*_3_	*f*_*e*_
3	*f*_1_+*f*_3_	*f*_2_	*f*_*e*_
4	*f*_2_+*f*_3_	*f*_1_	*f*_*e*_
5	*f*_1_+*f*_2_+*f*_3_	*f*_*e*_	$f_{e^{\prime }}$

This is a total of *B*_3_=5 cases. *f*_*e*_ and $f_{e^{\prime }}$ are the proportions of erroneous sequences

Let *p*_*ki*_ be the *i*-th expected frequency for case *k*. Assume the observed frequencies of the sub-sequences in a window *w*∈*W* follow a multinomial distribution. The likelihood value for the window *w*, (*L*(*w*)), is computed as follows:

$${\begin{aligned} &L(w)\\ &=\sum_{k} prob (\text{top } {n} \text{ observed frequencies in window } {w} | \text{case } {k}) \text{ prob}(\text{case } {k}) \\ & =\sum_{k}mult(g_{w1},g_{w2},...,g_{wn};n,p_{k1},p_{k2},...,p_{kn}) prob(\text{case}\ {k}) \\ & \propto \sum_{k} {\left(\prod_{i=1}^{n}{(p_{ki})^{g_{wi}}}\right) prob(\text{case}\ {k})} \end{aligned}} $$

The probability of the case *k* (i.e. *prob*(case *k*)) is estimated by the following equation:

$$\begin{aligned} prob(\text{case } {k}) & \approx \frac{1}{|W|}\sum_{w \in W} {Prob(\text{case } {k} | \text{window } {w})} \\ & \approx \frac{1}{|W|}\sum_{w \in W} {\frac{\prod_{i=1}^{n}{(p_{ki})^{g_{wi}}}} {\sum_{k}{\left(\prod_{i=1}^{n} {(p_{ki})^{g_{wi}}}\right)}}} \end{aligned} $$

And the overall log-likelihood value (*logL*) for all the windows *w*∈*W* is:

$$logL = \sum_{w \in W} {log(L(w))} $$

The optimal values of $f_{1},f_{2},...,f_{n},f_{e},f_{e^{\prime }}$ are computed such that the overall log-likelihood value (*logL*) is maximum. In practice, the following constraints are used: $f_{1} \geq f_{2} \geq \cdots f_{n} \geq f_{e} \geq f_{e^{\prime }}$ and *f*_*e*_≤*b*, where *b* is an upper limit for the frequency of an erroneous subsequence. The estimated sample proportions are the optimal values of *f*_1_,*f*_2_,...,*f*_*n*_. The time complexity is: *O*(*B*_*n*_∗*n*∗|*W*|), where *B*_*n*_ is the *n*-th Bell number, *n* is the number of haplotypes, and |*W*| is the number of windows.

### Reconstruction of haplotype sequences

The next step is to reconstruct the haplotype sequences according to the sub-sample proportions estimated in the previous step. We assume that each sub-sample is generated from a unique haploid sequence (i.e. haplotype). If we can identify the corresponding sub-sequence of each haplotype for every sliding window, then the haplotype sequences can be reconstructed by combining the sub-sequences from all the windows. However, in practice, it is not obvious, because the real sub-sequences are usually mixed with erroneous sub-sequences caused by sequencing errors. Moreover, multiple haplotypes may share the same sub-sequence and the observed frequencies of the sub-sequences may deviate from expectation at some positions.

A dynamic programming approach was used to reconstruct multiple haplotype sequences simultaneously, by considering all the cases for each window, and choosing the best arrangement with the maximum likelihood value.

Consider a sliding window *w*∈*W* and the top-*n* most frequent sub-sequences (i.e. *t*_*w*1_,*t*_*w*2_,...,*t*_*wn*_) in the window. Since each haplotype can generate one sub-sequence, there are *n*^*n*^ possible cases to generate *n* different sub-sequences by *n* haplotypes (considering that multiple haplotypes can generate the same sub-sequence and some sub-sequences can be erroneous), and each case will lead to a different set of expected frequencies of the sub-sequences. Table 7 lists all 27 possible cases and the expected frequencies of the sub-sequences when *n*=3.

**Table 7:** There are a total of 27 cases for generating 3 sub-sequences by 3 haplotypes

	Haplotypes which generate the sub-sequences			Expected frequencies		
Case	subseq1	subseq2	subseq3	subseq1	subseq2	subseq3
1	*h*_1_	*h*_2_	*h*_3_	*f*_1_	*f*_2_	*f*_3_
2	*h*_1_	*h*_3_	*h*_2_	*f*_1_	*f*_3_	*f*_2_
3	*h*_2_	*h*_1_	*h*_3_	*f*_2_	*f*_1_	*f*_3_
4	*h*_2_	*h*_3_	*h*_1_	*f*_2_	*f*_3_	*f*_1_
5	*h*_3_	*h*_1_	*h*_2_	*f*_3_	*f*_1_	*f*_2_
6	*h*_3_	*h*_2_	*h*_1_	*f*_3_	*f*_2_	*f*_1_
7	*h*_1_ & *h*_2_	*h*_3_	Erroneous	*f*_1_+*f*_2_	*f*_3_	*f*_*e*_
8	*h*_3_	*h*_1_ & *h*_2_	Erroneous	*f*_3_	*f*_1_+*f*_2_	*f*_*e*_
⋯	⋯	⋯	⋯	⋯	⋯	⋯
26	Erroneous	*h*_1_ & *h*_2_ & *h*_3_	Erroneous	*f*_*e*_	*f*_1_+*f*_2_+*f*_3_	$f_{e^{\prime }}$
27	Erroneous	Erroneous	*h*_1_ & *h*_2_ & *h*_3_	*f*_*e*_	$f_{e^{\prime }}$	*f*_1_+*f*_2_+*f*_3_

*h*_*i*_ represents that the sub-sequence is generated from haplotype *i*, and ’erroneous’ represents the erroneous sub-sequences. *f*_*i*_ is the estimated proportion of sample *i*, and $f_{e},f_{e^{\prime }}$ are the proportions of erroneous sub-sequences

Define *A*(*w*,*k*)=(*t*_1_,⋯,*t*_*n*_) as an assignment of the haplotypes to the sub-sequences in sliding window *w* when case *k* is considered (i.e. *i*-th haplotype generates sub-sequence *t*_*i*_,1≤*i*≤*n*). For example, as shown in Table 7, for *n*=3 and case 7, *A*(*w*,7)=(*t*_*w*1_,*t*_*w*1_,*t*_*w*2_) (i.e. the observed sub-sequence with the highest frequency in window *w* is generated from both the first and the second haplotypes, while the observed alignment with the second highest frequency is generated from the third haplotype).

Define *δ*(*A*(*w*,*k*),*A*(*w*^′^,*k*^′^)) as the compatibility between two assignments *A*(*w*,*k*)=(*t*_1_,⋯,*t*_*n*_) and *A*(*w*^′^,*k*^′^)=(*t*^′^_1_,⋯,*t*^′^_*n*_) and *δ*(*A*(*w*,*k*),*A*(*w*^′^,*k*^′^))=1 if, for all 1≤*i*≤*n*, two sub-sequences *t*_*i*_ and *t*^′^_*i*_ are exactly the same in their overlapped region. Mathematically, if the window size is *d*, the two windows overlap *l* bases, and window *w* is before window *w*^′^,

$$\delta(A(w,k),A(w^{\prime},k^{\prime})) \,=\,\! \left\{\begin{array}{ll} 1 & \text{if}\ t_{i}[d\,-\,l\,+\,1\! \cdots d] \,=\, {t^{\prime}}_{i}[1 \!\cdots l] \forall i \\ 0 & \text{otherwise} \end{array}\right. $$

We begin from a starting window *w*_*s*_∈*W* and consider all possible *n*^*n*^ assignments in *w*_*s*_. Then we consider the left and the right windows besides *w*_*s*_, and continue until all the windows have been considered. The optimal reconstruction of *n* haplotypes is the set of compatible assignments for all the windows with the maximum log-likelihood value. The following dynamic programming approach is used to compute the optimal compatible assignments for all the windows.

Given a starting window *w*_*s*_∈*W*, define *ζ*(*k*_*s*_,*k*_*t*_,*w*_*t*_), where *w*_*t*_∈*W*,1≤*k*_*s*_,*k*_*t*_≤*n*^*n*^, as the maximum log-likelihood value of the optimal compatible assignments for the consecutive windows from *w*_*s*_ to *w*_*t*_ with assignment *A*(*w*_*s*_,*k*_*s*_) in window *w*_*s*_ and assignment *A*(*w*_*t*_,*k*_*t*_) in window *w*_*t*_. If *s*<*t*, the assignment is proceeded from left to right, while if *t*<*s*, the assignment is proceeded from right to left.

Without loss of generality, considering the situation that the haplotype assignment is proceeded from left to right, the recursive formula of *ζ*(*k*_*s*_,*k*_*t*_,*w*_*t*_) is defined as:

$${\begin{aligned} \zeta(k_{s},k_{t},w_{t})\!&=\!\underset{\underset{\delta\left(A\left(w_{t-1},k\right),A\left(w_{t},k_{t}\right)\right)=1}{k\ \text{such that}}}{\max} \left(\zeta(k_{s},k,w_{t-1}) + log(like(w_{t},k_{t}))\right) \end{aligned}} $$

where *like*(*w*_*t*_,*k*_*t*_) is the likelihood value of the observed frequencies of the sub-sequences in window *w*_*t*_ when assignment *A*(*w*_*t*_,*k*_*t*_) is selected.

Let *q*_*ki*_ be the *i*-th largest expected frequency for case *k*.

$$\begin{aligned} like(w_{t},k_{t}) & = mult(g_{w_{t} 1},g_{w_{t} 2},\cdots,g_{w_{t} n};n,q_{k_{t} 1},q_{k_{t} 2},\cdots,q_{k_{t} n}) \\ & \propto \prod_{i=1}^{n} {(q_{k_{t} i})^{g_{w_{t} i}}} \end{aligned} $$

Therefore,

$$\zeta(k_{s},k_{t},w_{t}) \propto \underset{\underset{\delta\left(A\left(w_{t-1},k\right),A\left(w_{t},k_{t}\right)\right)=1}{k\ \text{such that}}}{\max} {\left(\zeta(k_{s},k,w_{t-1}) + \sum_{i=1}^{n} {g_{w_{t} i} log(q_{k_{t} i})}\right)} $$

In order to increase the accuracy of the haplotype reconstruction, we reconstruct the haplotypes starting from a relatively reliable window $w_{\hat {s}}$ with much dissimilarity between the haplotypes. When *n*=3, we locate the window $w_{\hat {s}}$ which have the greatest value of likelihood value for the case when each haplotype is assigned to different sub-sequence. Let the first and the last window on the haplotype region be *w*_1_ and *w*_*last*_. The haplotypes are reconstructed in both directions from the window $w_{\hat {s}}$ to the beginning and to the ending of the haplotypes, respectively. Considering the different case $k_{\hat {s}}$ for the starting window $w_{\hat {s}}$, the log-likelihood value of the optimal set of compatible assignments for the whole haplotype region is:

$$\max_{k_{\hat{s}}} \left(\max_{k_{1}} (\zeta(k_{\hat{s}}, k_{1}, w_{1})) + \max_{k_{last}} (\zeta(k_{\hat{s}}, k_{last}, w_{last}))\right) $$

Since *k*_*s*_ and *k*_*t*_ have *n*^*n*^ possible values (where *n* is the number of haplotypes), the overall time complexity of the method is: *O*(*n*^2*n*^∗|*W*|). The method explores all the possible cases and is an exact algorithm. The time is growing exponentially with the number of haplotypes. For higher number of haplotypes, a heuristic approach should be developed accordingly.

## Abbreviations

*B*_*n*_: *n*-th of the Bell numbers
HMM: Hidden Markov Model
JC: Jukes and Cantor model
N50: A weighted median statistic such that 50% of the entire assembly is contained in contigs longer than or equal to this value
